# The reproducibility of COVID-19 data analysis: paradoxes, pitfalls, and future challenges

**DOI:** 10.1093/pnasnexus/pgac125

**Published:** 2022-08-23

**Authors:** Clelia Di Serio, Antonio Malgaroli, Paolo Ferrari, Ron S Kenett

**Affiliations:** Vita-Salute San Raffaele University, UniSR, Milan, Italy; University Centre of Statistics in the Biomedical Sciences CUSSB, UniSR, Milan, Italy; Biomedical Faculty, Università della Svizzera Italiana, Lugano, Switzerland; Vita-Salute San Raffaele University, UniSR, Milan, Italy; Biomedical Faculty, Università della Svizzera Italiana, Lugano, Switzerland; Ente Ospedaliero Cantonale, Lugano, Switzerland; Clinical School, University of New South Wales, Sydney, Australia; KPA,Samuel Neaman Institute, Technion, Haifa, Israel; University of Turin, Turin, Italy

## Abstract

In the midst of the COVID-19 experience, we learned an important scientific lesson: knowledge acquisition and information quality in medicine depends more on “data quality” rather than “data quantity.” The large number of COVID-19 reports, published in a very short time, demonstrated that the most advanced statistical and computational tools cannot properly overcome the poor quality of acquired data. The main evidence for this observation comes from the poor reproducibility of results. Indeed, understanding the data generation process is fundamental when investigating scientific questions such as prevalence, immunity, transmissibility, and susceptibility. Most of COVID-19 studies are case reports based on “non probability” sampling and do not adhere to the general principles of controlled experimental designs. Such collected data suffers from many limitations when used to derive clinical conclusions. These include confounding factors, measurement errors and bias selection effects. Each of these elements represents a source of uncertainty, which is often ignored or assumed to provide an unbiased random contribution. Inference retrieved from large data in medicine is also affected by data protection policies that, while protecting patients’ privacy, are likely to reduce consistently usefulness of big data in achieving fundamental goals such as effective and efficient data-integration. This limits the degree of generalizability of scientific studies and leads to paradoxical and conflicting conclusions. We provide such examples from assessing the role of risks factors. In conclusion, new paradigms and new designs schemes are needed in order to reach inferential conclusions that are meaningful and informative when dealing with data collected during emergencies like COVID-19.

## Background

Our retrospective inspection of COVID-19 data analysis, named for its unprecedented volume as the “COVID-torrent” (1), joins the multifaceted experience and perspective of statisticians, epidemiologists, and clinicians. The objective is to balance challenges and pitfalls of data acquisition and data analysis in the context of public healthcare and clinical decision-making. We refer to three phases of the COVID-torrent. Specifically, we ask whether the large amount of generated COVID-related data translated into solid knowledge about this viral infection, the disease, and its treatment. The answer is that the COVID-torrent stands out for its dismal outcome (2). In part, this can be attributed to the complexity of the COVID-19 pathology. We also need to acknowledge that COVID related data acquisition and analysis was not tailored to provide a common pathway to aggregate very different data sets. One can say that we have been “Drowning in data and starving for information” (3). The silver lining is that the COVID-19 pandemic provided a fundamental testing ground to implement more effective data collection, analysis, modeling, and interpretation tools, across the entire world. In addressing this challenge, we invoke the importance of an information quality (InfoQ) framework that provides an approach to assess available data and its analysis, in the context of specific goals (4). Assessing the quality of the information generated by COVID-19 data and its analysis helps to turn numbers into information.

## Covid-19 Publication Flood

Since the COVID-19 pandemic started, in February 2020, we witnessed an unprecedented effort to find answers on how to contain the virus spread, how to deal with its consequences and how to find a breakthrough treatment. We identify three phases in the related publication flood. In a first phase, the focus was mainly on COVID-19 prevalence, incidence, and transmission rate. Viral load was connected to disease severity, treatment combinations, and efficacy of the different therapeutic protocols. In the second phase, the focus shifted to the analysis of COVID-19 herd immunity, antibodies levels, and asymptomatic characterization. In a third phase, most of the attention was captured by different available vaccines, vaccination protocols and their therapeutic efficacy, and also by predictions of new infection waves and the genesis of new variants. This holistic perspective has been discussed in the recent literature (5).

Comparative testing of different vaccines, in different subgroups and countries, was effective in selecting the most effective vaccines and those less prone to side effects. Determining the optimal number of vaccine doses and how long their protection is lasting, their efficacy against the different COVID-19 variants, figuring out if vaccinated people could still become infected or transmit infections and determining the best interval for the vaccination boosts while understanding if it is useful to vaccinate those who already had the disease, have all proven to be complex questions. Leading publishers (http://acdc2007.free.fr/drowningcovidpapers.pdf) got involved in a controversy between a general effort to make access to COVID-19 related papers free, with no embargo time, and the need of preserving papers quality through the conventional review processes. In 2020 alone, more than 400,000 COVID-19 related publications have been collected by the COVID-19 Open Research Dataset (6). One of the largest collection of publications repository, the COVID-19 Open Research Dataset (CORD-19), includes over 500,000 scholarity articles. In response to this flood of publications, several initiatives were implemented for selecting robust and generalisable papers. The lack of common criteria and qualitative requisite to standardize the data made this difficult. Some spontaneous and independent projects aimed at finding a list of common COVID-19 records. Examples include the COVID-19 Real World Data (RWD) Data Elements Harmonization Project (7), and international organizations and agencies (e.g. Eurostat, WHO, ECDC, and CDC). However, it seems that these did not provide effective support to public health local institutions and to the harmonization of health indicators related to Coronavirus.

In the COVID-19 pandemic, the collected data were expected to provide useful information, in a very short time (8). This urgency lead to applications of artificial intelligence algorithms in order to retrieve data-driven insights in apparent conflict with the need of journals to ensure scientific rigor (9). This phenomena was a divergence from the fundamental approach to science which is based on critical reasoning and statistical thinking. In general, science seeks to differentiate sources of noise from reproducible true effects. This approach requires time and does not necessarily meet the requirement of urgency. Indeed, it is difficulat to find straightforward approaches that describe, in an interpretable way, the complexity of transmission mechanisms from observational clinicaly available information. Complex integrated approaches are needed in order to combine different sources of information. A statistical thinking perspective is needed to balance modeling difficulties and generate valid inference and reliable predictions. On the other hand, artificial intelligence algorithms have been used for selecting prediction models that integrate the clinical symptoms and features of patients with COVID-19 (10). Moreover, epidemiological models (SEIR) (11) and physics-based approaches based on Brownian motions (12) were used to investigate different scenarios and public health interventions aimed at minimizing the impact of outbreaks. These approaches provided decision makers the ability to test the impact of different control strategies. However, these modeling approaches often proved inconsistent with data showing nonlinear behaviors, sparsity patterns and partial collection.

In order to advance knowledge, all these considerations need to be matched by an in-depth discussion on data and analysis methods. On the one hand, there were important deficiencies in public health systems such as (i) slow response of public health organizations in providing guidelines for uniform epidemic parameters definition and data-sharing; (ii) data being poorly monitored at crucial crossroads, like early outbreak or new mutant strains; (iii) different and disorganized criteria for data collection; and (iv) lack of an open science data system. On the other hand, there has been an over-reliance on data-driven methods as tools that shed light on the pandemic, sometimes at the detriment of statistical thinking. For example, in order to monitor both infection and immunity over time, sample-based surveillance schemes, built on statistical principles of population representation are recommended. This is in contrast to non-probability convenience sampling that was widely implemented, all over the world.

The outbreak of COVID-19 took the modern world by surprise and provided an opportunity to review lessons from past infections and pandemics. In the next section we provide an example.

## A Historical *Déjà vu*

The history of biomedical discoveries teaches us that translating information into knowledge has always been a major challenge in clinical research. It took almost 7 years to understand that Albert Sabin's oral polio-vaccine made with attenuated poliovirus, was with almost no side effects (13), and much more effective than the Jonas Salk’s vaccine introduced in 1955. The scepticism met by Sabin to his discovery was due to the fact that it implied abandoning an already effective prophylactic treatment. It related to the inability to convert available clinical data to information and predictions. This forced Albert Sabin to test his vaccine in Russia, before it became acceptable worldwide. It took until 2006 to have the member states of the world health assembly on poliovirus “elimination” for polio-endemic to declare their commitment to interrupt transmission of two of the three wild-type poliovirus using appropriate monovalent oral polio vaccines. Indeed, we distinguish between disease elimination and disease eradication. In case of both poliomyelitis and measles we refer to “elimination” of the infection. Elimination occurs when incidence of infection caused by a specific agent shrinks to zero as a consequence of deliberate and common efforts. However, continued measures to avoid re-establishment of transmission are needed. The term “eradication” applies when we reach of a permanent zero incidence of infection worldwide and intervention measures are no longer required. This seems to be the case of smallpox with ring vaccination. With the rapid emergence of SARS-CoV-2 variants that can escape natural or vaccine-induced immunity, eradication or elimination of COVID-19 is unlikely to occur.

Considering COVID-19 under control, implies the reduction of disease incidence, prevalence, morbidity, or mortality to an endemic level as a result of deliberate efforts that allow to accomplish “herd immunity.” Indeed, herd immunity, induced either by natural infection or vaccination, achieves a threshold immunity at the population level that cuts the net transmission of the disease. This threshold immunity can protect the largest proportion of inhabitants of a given geographical area within a certain timeline. Whether the effect is lasting depends on the duration of individual-level natural or vaccine-induced immunity. There are several reasons why COVID-19 herd immunity is less likely to be reached. These include poor vaccine acceptance, emergence of new variants that are more transmissible and circumventing global vaccination programs.

Therefore, control of an infectious disease is not achieved through vaccine coverage alone, but needs to be approached in its entirety. This includes “data-driven” policies for identification of outbreak patterns, risk profiling of susceptible populations, construction of a surveillance statistical sampling, scheme data-integration techniques to integrate mobility and health data with clinical information. The common denominator is all these approaches is data quality, information quality and statistical thinking. The next section expands on the informtion quality framework.

## Information Quality

Some of the important challenges that emerged from the COVID-19 experience are reflected by three questions: (i) Can we improve and standardize data acquisition and database construction to include all relevant data and exclude meaningless, not accurate, or unreliable sources? (ii) Can we make data acquisition more flexible and dynamic, so that we can quickly address target-oriented issues that may arise over time, and speed up the achievement of insight on the underlying phenomen? (iii) Can statistics provide a new architecture of analyses to improve the gap between “large data” and “large knowledge?" or "information quality?"

Generating new knowledge is supported by structured information quality (InfoQ). InfoQ is defined as “the potential of a dataset to achieve a specific (scientific or practical) goal using a given empirical analysis method” (4). The InfoQ framework consists of four components: (i) Goal, (ii) Utility, (iii) Data, and (iv) Analysis Method and eight dimensions: (1) Data Resolution, (2) Data Structure, (3) Data Integration, (4) Temporal Relevance, (5) Chronology of Data and Goal, (6) Generalizability, (7) Operationalization, and (8) Communication. These components and dimensions determine the InfoQ provided by a specific study. InfoQ, generated from COVID-19 data, is affected by many limitations such as poor study design, incomplete data, poor data resolution, ineffective data-integration, and weak generalizability. These barriers prevent the performance of a meaningful analysis. Poor InfoQ can be due to conditions of data acquisition and data analysis. In emergency conditions, operationalization and generalization of findings drive data collection needs that account for a wide range of possible sources of bias.

## Data Quality, Sources of Biases, and COVID-19 Paradoxes

When dealing with large data, collected without proper study design, one major issue is the possible impact of several types of bias. Example include selection bias and nonresponse bias. These may affect results in a paradoxical way, leading to confounding bias. One major source of bias, that affects big data in general and COVID-19 data in particular, is related to “non probability” sampling procedures. Such data collection weakens the resulting statistical inference. Indeed, probability sampling is based on designed variables, known in advance, for all units that define the population frame. In non-probability samples, the inclusion probability is unknown. Indeed, bad sampling methods, cannot be fixed by increasing the size of the sample. The population size, *N*, plays the role of a “magnifying lens” that amplifies bias of nonprobability sampling and can produce highly erroneous inferential conclusions. It can be shown (14) that under non probabilistic sampling, the MSE (mean square error) that measures the amount of error in the statistical model increases with sample size. In such settings one needs to implement alternative statistical analysis methods, such as the various weighting approaches. One option is the use of propensity score to construct weights that account for bias in the nonprobability sample. New statistical perspectives have been recently proposed in analyzing large data from nonprobability samples (15). This method overcomes possible bias using a standard logistic regression that relies on a priori assumptions of functional relations among observed variables. Another new approach is based on the use of Bayesian Network to estimate propensity score (15).

However most published papers still apply standard statistical techniques, even with nonprobability sampling, thus ignoring possible sources of bias. Aside from selection bias and nonresponse bias, confounding bias can induced to erroneous interpretation of the role of covariates, and paradoxical conclusions. This has been widely seen in the interpretation of risks factor in COVID-19 data. Indeed, standard adjustment methods for covariates, like the use of odds ratio, lead to several misleading paradoxes including the “obesity paradox,” “ACE-inhibitors paradox,” and the “smoking paradox.” Most of these paradoxes can be attributed to unaccounted sources of variability that produce confounding effects. These include, among others, erroneous imputation of missing values, lack of uniform definition of variables in databases, and unaccounted interaction effects of age unmeasured degree of “severity.” Moreover, data from different COVID-19 waves cannot be easily merged and compared not only because viral variants could be biologically different in their infection and pathogenetic mechanisms, but also because the time exposure to therapeutic agents, and the introduction of new treatments could affect the clinical outcome.

For instance, the role of obesity, a well-known risk factor in cardiovascular diseases, was evaluated in many COVID-19 research papers (16). Obesity was shown to be a risk factor during the first wave of COVID-19 but also claimed to be “protective,” or without effects (17). These contrasting results arise from many potential caveats such as: (1) Different selection criteria to evaluate obesity. The body mass index (BMI) is a fundamental tool, but not sufficient per se to diagnose obesity without a clinical evaluation. Databases should be tagged when this clinical diagnosis is met, (2) Criteria and cut off values for clinical diagnosis of obesity variy among countries and depend on ethnicity, (3) Missing BMI values can be outcome related. In severely ill patients, weight and particularly height cannot be easily measured, in urgency conditions. This represents a censoring effect rather than a “missing at random” condition and hence cannot be corrected by simple imputation. Another paradox refers to the widely discussed low risk of developing COVID-19 in habitual smokers. Many papers reported no significant correlation between active smoker and severity in COVID-19 (18), dismissing the effect of smoking on respiratory functions. However, it can be shown that smoking variables are often poorly measured (0/1 variables). Such data comes mainly from hospitalized severe patients, in age groups with no-longer smokers. The paradox results in a selection bias. Similar considerations hold in relation to the ACE-inhibitor paradox, an important discussion topic during the first COVID-19 phase (19). At the beginning, an increased risk of COVID-19 in patients treated with ACE-inhibitors was reported. Later on, the opposite was demonstrated, i.e. a protective effect of ACE-inhibitor medications (20). The confounding effect of age on all covariates was not appropriately accounted for in most of the literature we reviewed.

These phenomena are classified as “false protectivity” (21, 22). They are due to biased measurements and ignore underlying data structure and data integration which are the second and third dimension to consider in the InfoQ framework. A lack of designed studies can have severe effects on treatment protocols.

Another issue is that data was not collected according to declared exposure factors, with a usual “control” definition. COVID-19 data cannot be framed within an observational retrospective design. As in many published papers, one has to wonder what are the proper “controls” in such a pandemic.

The general nature of “controls” depends on the type of study considered and the research hypothesis. It should be carefully selected. According to historical literature (23), selecting controls should be performed on principle of comparability and in particular: (i) all comparisons should be considered within the “study base” which means that controls for hospitalized COVID-19 patients should be nonhospitalized COVID-19 patients; (ii) deconfounding: dependence among different levels of exposures should be considered in affecting disease risk to avoid distortions; and (iii) comparable accuracy.

Unfortunately, information of nonhospitalized patients was not available since universal nasopharyngeal rt-PCR screening of people with no or very mild symptoms has not been systematically performed. Thus, most publications used controls randomly sampled from primary healthcare database without any knowledge on disease status of controls.

The majority of studies on COVID-19 are case series and need to be analyzed as such. A proper analysis should investigate patient profiles within a complex dependence network structure in order to evaluate “net” effects of risk factors toward a causal inferential approach. Indeed, a data-driven perspective needs to integrate prior-clinical knowledge and cannot entirely rely on algorithms. Machine learning approaches may fail in describing the relation between COVID-19 risks factors, since they are intrinsically investigating “correlative” structure only (24). Any “causal” conclusion should be anchored in a validation and sensitivity analysis perspective to ensure reproducibility and replicability (25).

Therefore, new analysis paradigms, for these types of data, need to be developed. These methods should be able to control for confounding bias, different data-integration protocols, data resolution and data structure, i.e. focus on the generation of InfoQ.

The impact of poor data quality on medical science has been widely discussed in the literature (26). However, nowadays the increasing availability of medical data and the changes occurred in data acquisition methods create new challenges in the data-quality paradigm versus more technical and ethical features such as (i) data-sharing, (ii) data-integration, and (iii) privacy.

## Data Quality, Data-Sharing, and Reproducibility

A paramount issue often disregarded is origin of persisting barriers in sharing clinical data. Indeed, data-sharing is not always positively perceived and the opinions and perspectives about it vary depending on the field of study. This should be at the top of the priority list. Data should be shared in a complete manner, possibly even when data acquisition is still running and information gathered. In biomedical science, data-sharing is fundamental since: (i) it allows reproducibility of research, (ii) it helps scientists use real-world data sets whenever well-designed studies are less available; and (iii) it promotes scientific work and progress by analyzing previous findings (27–29).

One famous example that highlights the enormous benefits of data-sharing refers to the selective COX-2 nonsteroidal anti-inflammatory drug, Rofecoxib. This drug was approved in 1999 by the US Food and Drug Administration (FDA) to treat rheumatoid arthritis, acute pain, and dysmenorrhea. The clinical data made available by the drug company (the VIGOR trial with 8,076 enrolled patients) indicated that it was superior to other painkillers. During data acquisition, an increased risk of serious heart problems was identified, but the manufacturer decided to hold the information and combined the results of VIGOR with results of another Rofecoxib trial. This downplayed this side effect (30). In the following years, many reports called attention to the increased risk of cardiovascular problems (31, 32), This forced the manufacturer, in 2004. to withdraw the drug and to admit it withheld information (33, 34). In 2007, the manufacturer announced the payment of $4.85 billion to end thousands of lawsuits, the largest drug settlement ever. This type of experience helped change biomedical research. In the recent decade, data-sharing in biomedical research became more prevalent, whereas data-sharing in other areas of research such as meteorology and economics have been common practice, for years.

**Fig. 1.**
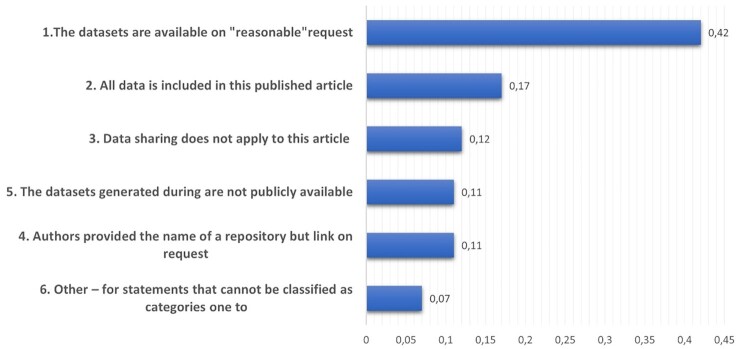
Categories of Data Availability Statements (DAS) in 3,416 published papers. Data drawn from (37).

The COVID-19 experience has shown how strong resistance to data sharing can dramatically slow fast acquisition of knowledge on a global disease. Several contributions have highlighted the lack of incentives to be the limiting step for data-sharing (35). Resilience to share data is connected to psychological and practical motivations that are part of the researchers’ aversion to share data. In a recent study (36) performed on a sample of 321 researchers comparing personal “attitude” and “intention” to data-sharing, the main impediments were the distrust in technological support and the fear of getting their scientific findings scooped. Most respondents reported a positive attitude toward data-sharing and said they are willing to share data with other researchers and support open science research. However, when testing how researchers trust the availability of an adequate system to share the data, less than one-third indicated that they were open to deposit the data of their published articles in such a data repository. These results suggest that researchers are less confident than what they declare in sharing data through current data repositories and communication tools. Similar considerations were confirmed when researchers’ compliance with their Data Availability Statement (DAS) was measured in 3,416 published papers (37), showing that only 17% of articles really made data publicly available from publication. It can be easily seen the difference between “published” data and “public” data, i.e. declaring that data are “available”, assumes many different meanings that prevents the readers from a straight and direct access to data, see Figure 1.

Aside of the aforementioned psychological reasons, resilience to share data might be related to technical “data quality” issues and to legal questions such as informed consent or ethics approval. Indeed, simply sharing data does not improve science unless the data is usable by the scientific community for building shared platforms enabling data-integration. Moreover, combining multiple data sources is a complex process that needs techniques to resolve inconsistencies in temporal structure, encoding and handling of missing values, inconsistencies in defining common variables, varying data resolutions, and nonstandardization of case definitions.

Defining common guidelines that determine how data should be analyzed to allow a meaningful comparison of results generated by different sources will enhance best practices to ensure data integrity and become a common practice.

## Data Quality, Data-Integration, and Statistical Perspectives

The COVID-19 experience showed us that improving the quality of inference and gaining further knowledge by cross-check and integration of data collected in heterogeneous clinical settings, such as hospitals, intensive care units, general and specialist health departments in both private and public sectors, has become a fundamental necessity. Effective data-integration produces statistical inference that is more robust and efficient with respect to options based on single data sources. The first obstacle in this is represented by data extraction. Implementing a bio-bank often implies merging electronic medical records with data from text-based reports (like clinical diary). This requires contextual understanding and interpretation from experts. For instance, throughout the COVID-19 pandemic, data formats not only differ across countries, but also within the same reporting sources, over time.

In the last two decades, there has been wide consensus about the strategic potential benefits in implementing tools that enhance data-integration in different disciplines. Data-integration is achieved by two main methods: *statistical matching* and *record linkage*. The main feature of these procedures can encounter important limitations in emergency conditions like a pandemic. An essential distinction between these two approaches derives from the type of data sources to integrate. *Statistical matching* is closer to the idea of “merging” different data sets. Being model-based provides joint information on variables and indicators collected through multiple sources. The main feature of *statistical matching* concerns the population of interest: although the units in the data sets can differ, these should come from the same population, and are usually not overlapping. A different perspective is to link information on same units. This is achieved by *record linkage* that allows to integrate data whenever units included in the data sets overlap. *Record linkage* cope with identical units, while *statistical matching*, handles “similar” units from the same population.

Many limitations to this process arise in pandemic contexts, mainly due to the lack of harmonization in electronic health records and in disease definition criteria. First, *statistical matching* is hampered by the lack of representativeness of COVID-19 patient characteristics that do not respond to common criteria and cannot refer to the same population. Moreover, *record linkage* cannot be used correctly in integrating COVID-19 data when missing values are present, or when databases are ambiguous in uniquely identifying patients. This is accentuated when there is a high amount of noise in patient information.

The above considerations explain why even the most elementary epidemiologic COVID-19 questions remain unanswered. The culprits are the poor reporting of infection and the unknown sampling designs. Consider the questions: What is the prevalence of the disease in the general population and in subpopulations? Are there specific clinical phenotypes that need to be treated differently? What is the real case-fatality rate? Are asymptomatic cases really infectious? How long does immunity last? In general, as mentioned above, nonprobability sampling affect representativeness of the target population and inference reuires enlarging the degree of generalizability. Data-integration methods vary according to the types of samples and the type of information to be combined. In the COVID-19 pandemic, data-integration often failed due to under- or over- or differential representativeness of the different data sets. Tools trained on such integrated data retain bias on specific subpopulations (38–40). Lack of, or nonhomogeneous representativeness of the different data sets, increases the difficulty in finding effective ways to improve the data analysis process with statistical tools. In addition, the lack of representativeness impedes the reproduction of results from independent sources, i.e. a poor “generalizability” issue. In general, data-integration, can help overcome two important limitations: (i) integrating the results from different sources of data (i.e. clinical and biological) from the same individual. This is essential when dealing with big data analysis in omics research, where genomics, epigenomics, transcriptomics, proteomics, and metabolomics data, need to be integrated; (ii) integrating information on fundamental covariates for understanding the course of a disease when coming from disjoint samples. Different levels of information can be combined leading to different methods for probability data-integration: a macro approach that describes multiple surveys by means of summary statistics, and a micro approach to enhance synthetic imputations. Recently, the possibility to combine randomized clinical trials and observational data is considered. In this context, two types of data-integration can be considered: horizontal and vertical. Horizontal integration aims at integrating different sources of data with large number of variables and small sample size. This often occurs in basic research and in genomic meta-analysis. Additionally, we might have multiple data types and variables on the same set of samples, what we can consider as “vertical integration.” This can be achieved both with unsupervised or supervised methods. Integrating data with a large number of variables requires variable selection methods for predictive modeling of outcomes, but there is still a gap to fill in the literature on variable selection for data-integration to control for sources of bias still retaining all information captured for finite population inference.

In the future, efforts in this direction might provide vaccine surveillance protocol based on a statistical sample of vaccinated subjects stratified by risks profiles, and monitoring protocols for immunological parameters longitudinally for the duration of immunity in general and in comorbidities, such as age, BMI, and other covariates.

## Data Quality, Privacy, and Data Protection

Big data analysis requires processing clinical information on patient characteristics, demographics, patient profiles data, biomarkers, and therapies. Apart from the technical obstacles discussed above, another hurdle is complying with the fundamental rights to privacy and data protection of patients, as for instance set out by the 2018 EU General Data Protection Regulation (GDPR), considered the toughest privacy and security law in the world (https://gdpr.eu/tag/gdpr/). Unspecified big data analysis disrupts the paradigm “consent or anonymity,” according to which the processing of medical data for research purposes requires either individual specific informed consent or anonymization of personal data processed for the research. For an informed consent to process health data to be valid, the consent must be voluntary, unequivocal, specific, and explicit. Obtaining such consent is resource intensive, both in terms of manpower and costs, given that consent often needs to be gathered from a large number of individuals who may not be readily accessible (or even alive). Anonymized personal information can be used to avoid the need for informed consent, because once the personal data are rendered irreversibly anonymous, the data subject is no longer identifiable. However, perfectly anonymous data, while protecting patients’ privacy, are likely to render the data less useful or even useless for big data analysis. For instance, an analysis on an irreversibly anonymized database, with only clinical data, would greatly protect the privacy of the patients, but reduce the utility to be linked to a biobank or other valuable datasets. On the other hand, the growing availability of potentially complementary data online increases the chances to reidentify particular individuals from a dataset of anonymized data. The ability of gaining approval for various forms of research, in the absence of individual consent, is to access different repositories. This is an option available by law in many countries. However, this route is further hampered by the applicable ethical regulation, which can be highly variable from country to country, from region to region, and even between various types of institution.

Obtaining meaningful consent or irreversibly anonymizing data is unlikely to be practical or possible for a great deal of data-intensive medical research. How to deal with the challenges of informed consent or the anonymize approach in the context of data-intensive medical research, within the GDPR framework on data protection, is becoming increasingly difficult and represents a crucial future challenge.

## The Need for a Common Language

Finally, the gap has been extreme between the amount of complex data daily communicated and the poor level of statistical literacy of the general public that left the door open to a communication style that could be defined as data-terrorism rather than real informative communication.

The COVID-19 pandemic reinforced negative attitudes—outside of professionally managed programmes—to expose common people and fragile patients to uncontrolled and incomplete information. The World Health Organization (WHO) claimed that the “infodemic” surrounding COVID-19 spread “just as quickly as the virus itself, with conspiracy theories, rumors, and cultural stigma all contributing to deaths and injuries.” The destructive power of misinformation in the era of global communication highlighted the need to contrast misinformation with education to distinguish reliable sources, what should not be confused with an attempt to censor information (41). Recent literature has been highlighting the perils of misinformation (42) that did not contribute to increase of health literacy on COVID-19, but rather of mistrust in medicine and public health systems.

There has been an unprecedented and continuous degree of misinformation on the COVID-19 pandemic that might have nullified the benefits of patients’ empowerment. Indeed, this lead many patients who are misinformed but take active participation to health-related decisions to meet poor choices not related to their own benefits. The large number of COVID-19 deaths related to vaccination refusal represent a dramatic example of this. Likewise, it has been often seen in the literature that there is a strong association between low literacy and poor disease outcome. For instance, for common disorders like hypertension that has a prevalence estimated between 26% and 31% in adults (1.39 billion in the world from ECDC estimates in 2020) and treated only in low proportion (between 15.5% and 17.4% of patients), low literacy and misinformation is associated with uncontrolled blood pressure in 45% of hypertensive patients (43). Thus, one should also consider *communication*, the eighth InfoQ dimension. To support this dimension, educational programs have been promoted by statisticians (44, 45) aiming to train journalists and communicators in meeting new challenges not only related to the search of accurate information and sources but also to deepen the overall level of statistical “numeracy” in the data journalism. A better understanding of informative contents in COVID-19 data, can support media in an appropriate communication of policy-based restrictions committing people to a collaborative effort toward preventive measures (46). A general need for improving overall scientific literacy is becoming crucially related to all biomedical disciplines (47).

**Fig. 2.**
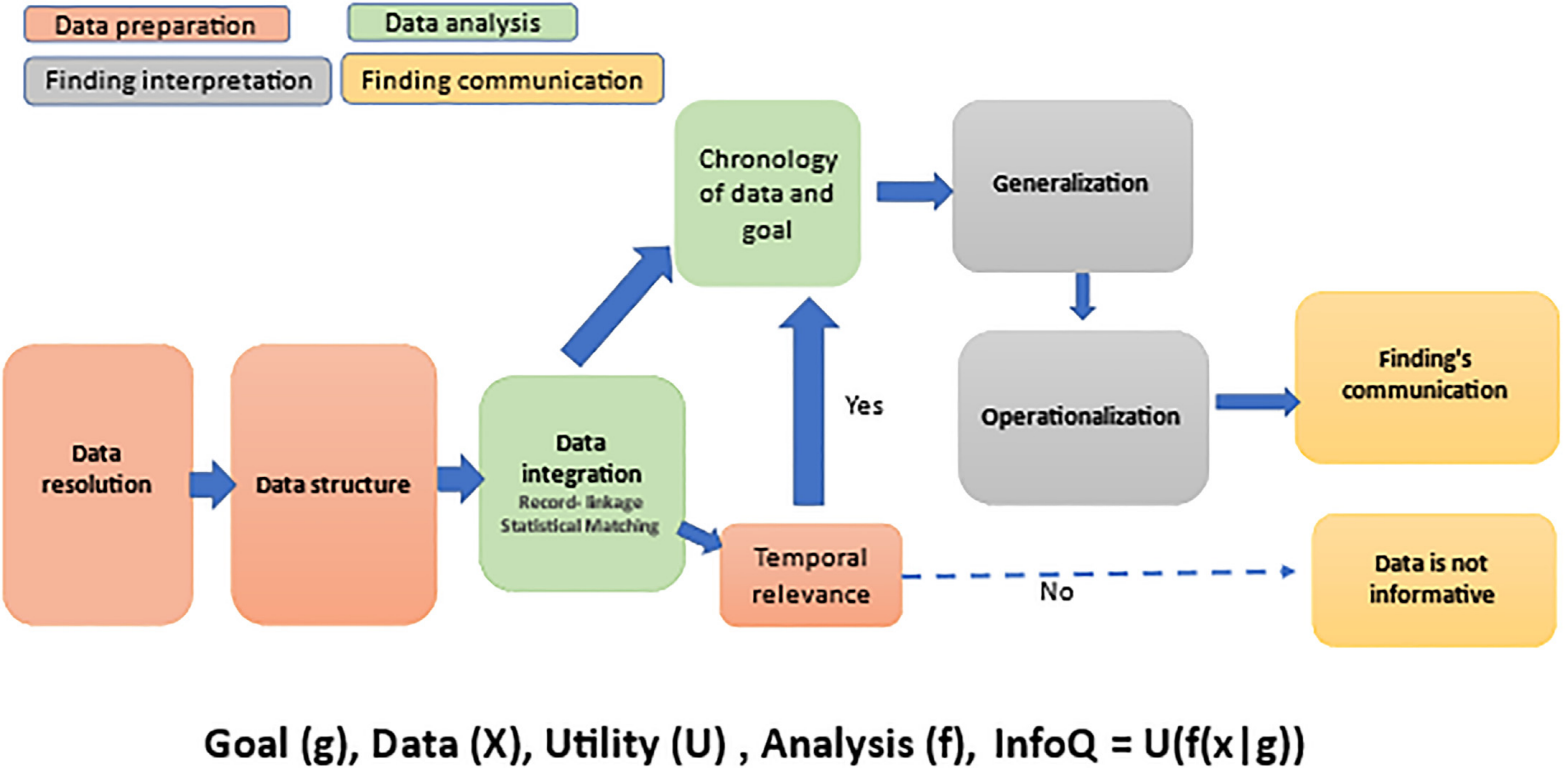
Comprehensive scheme for InfoQ, data-integration and communication issues.

Figure 2 merges in a schematic flow-chart the components, dimensions and features of InfoQ, data-integration and findings communication to achieve better level of preparedness of the public health system to any type of infectious or chronic disease.

## Conclusions

The COVID-19 pandemic has brought to the fore a series of paradoxes and pitfalls related to data quality and analysis. This impacts the scope and role of statistical analysis in assisting better management of the challenges of future global threats. Thus, our perspective calls to enhance readiness levels to future challenges. These could be topics relevant to policy-makers or healthcare organizations, like loss of efficacy of antibiotics, transportation congestion, traffic management in smart cities, and environmental issues like global warming.

In the initial phase, COVID-19 data were acquired under strong pressure from the public and politicians. The urgent goal was to establish policies and treatment protocols for an unprecedented condition with high mortality that exploded without warning. This resulted in nontargeted and inconsistent data collection with many unconfirmed results leading to inappropriate public health decisions. Today, given the considerable amount of data accumulated, it is possible to look back and find ways to recover this information globally and to develop more plausible and rigorous analysis protocols and integrated analysis models (5). This review was prepared to help advance this direction, and based on COVID-19 experience, help establish a general consensus among health specialists on how to address future pandemic data acquisition, categorization, integration, and analysis.
